# Kaposi’s sarcoma-associated herpesvirus seropositivity is associated with parasite infections in Ugandan fishing communities on Lake Victoria islands

**DOI:** 10.1371/journal.pntd.0007776

**Published:** 2019-10-16

**Authors:** Angela Nalwoga, Emily L. Webb, Belinda Chihota, Wendell Miley, Bridgious Walusimbi, Jacent Nassuuna, Richard E. Sanya, Gyaviira Nkurunungi, Nazzarena Labo, Alison M. Elliott, Stephen Cose, Denise Whitby, Robert Newton

**Affiliations:** 1 MRC/UVRI and LSHTM Uganda Research Unit, Entebbe, Uganda; 2 London School of Hygiene & Tropical Medicine, London, United Kingdom; 3 Viral Oncology Section, AIDS and Cancer Virus Program, Leidos Biomedical Research, Inc., Frederick National Laboratory for Cancer Research, Frederick, Maryland, United States of America; 4 College of Health Sciences, Makerere University, Kampala, Uganda; 5 University of York, York; United Kingdom; George Washington University School of Medicine and Health Sciences, UNITED STATES

## Abstract

We investigated the impact of helminths and malaria infection on Kaposi’s sarcoma associated herpesvirus (KSHV) seropositivity, using samples and data collected from a cluster-randomised trial of intensive versus standard anthelminthic treatment. The trial was carried out in 2012 to 2016 among fishing communities on Lake Victoria islands in Uganda. Plasma samples from 2881 participants from two household surveys, the baseline (1310 participants) and the final (1571 participants) surveys were tested for KSHV IgG antibody responses to K8.1 and ORF73 recombinant proteins using ELISA. The baseline survey was carried out before the trial intervention while the final survey was carried out after three years of the trial intervention. Additionally, a subset sample of 372 participants from the final survey was tested for IgE, IgG and IgG4 antibody concentrations to *S*. *mansoni* adults worm antigen (SWA) and *S*. *mansoni* egg antigen (SEA) using ELISA. Infection by helminths (*S*. *mansoni*, *N*. *americanus*, *T*. *trichiura* and *S*. *stercoralis*) was diagnosed using real-time PCR, urine circulating cathodic antigen (CCA) and stool microscopy (Kato-Katz method) while malaria infection was diagnosed using microscopy. We analysed the relationship between helminth and malaria infections and KSHV seropositivity using regression modelling, allowing for survey design. At baseline, 56% of the participants were male while 48% of the participants were male in the final survey. The most prevalent helminth infection was *S*. *mansoni* (at baseline 52% and 34% in the final survey by microscopy, 86% by CCA and 50% by PCR in the final survey). KSHV seropositivity was 66% (baseline) and 56% (final survey) among those 1–12 years and >80% in those 13+ years in both surveys; malaria parasitaemia prevalence was 7% (baseline) and 4% (final survey). At baseline, individuals infected with *S*. *mansoni* (detected by microscopy) were more likely to be KSHV seropositive (aOR = 1.86 (1.16, 2.99) p = 0.012) and had higher anti-K8.1 antibody levels (acoefficient = 0.03 (0.01, 0.06) p = 0.02). In the final survey, *S*. *mansoni* (by microscopy, adjusted Odds Ratio (aOR = 1.43 (1.04–1.95), p = 0.028) and malaria parasitaemia (aOR = 3.49 (1.08–11.28), p = 0.038) were positively associated with KSHV seropositivity. Additionally, KSHV seropositive participants had higher *S*. *mansoni*-specific IgE and IgG antibody concentrations in plasma. Furthermore, HIV infected individuals on cART were less likely to be KSHV seropositive compared to HIV negative individuals (aOR = 0.46 (0.30, 0.71) p = 0.002). *Schistosoma species* skew the immune response towards Th2 and regulatory responses, which could impact on KSHV reactivation if co-infected with both organisms.

## Author summary

Kaposi’s sarcoma associated herpesvirus (KSHV), the causative agent of Kaposi’s sarcoma cancer, varies geographically. KSHV infections are highest in sub-Saharan Africa, with Uganda having the highest prevalence reported to date. Infection with KSHV is lifelong with an intermittent revival of the virus, leading to viral spread. In this study, we show that infection with *Schistosoma mansoni* and malaria parasites is associated with being infected or exposed to KSHV. These parasite infections interfere with the proper functioning of the immune system to control viral infections. Although not shown in the current study, these parasite infections might lead to reactivation of KSHV in infected people increasing the likelihood of having detectable KSHV antibodies. Consequently, this viral reactivation may increase the spread of KSHV in sub-Saharan Africa.

## Introduction

The prevalence of Kaposi’s sarcoma associated herpesvirus (KSHV), also known as human herpesvirus 8 (HHV8), varies geographically, unlike that of other herpesviruses which are ubiquitous [1–3]. Uganda has a high prevalence of KSHV [4, 5] and a high incidence of Kaposi’s sarcoma (KS) [6, 7]. The incidence of KS rises dramatically among immunocompromised individuals [8–10]; immunosuppression has been implicated in the reactivation of KSHV and the progression of KS [9, 11].

Co-infection with helminths has been shown to modulate immune responses to other infections and vaccines [12–14]. Chronic infection with *Schistosoma* is characterised by the production of IL4, IL5 and IL13 cytokines, typical of a T helper (Th) type 2 response and IL10, a regulatory cytokine [15, 16]. The skewed immune response to a Th2 and regulatory response may impair the T helper (Th) 1 response, vital for control of viral infections [17–19]. The impact of *Schistosoma* co-infection on herpesviruses and other viruses has been demonstrated in animal models, where *Schistosoma mansoni* infection led to IL4-mediated reactivation of murine herpesvirus 68 and M2 macrophage polarization [17, 18].

Our group has documented associations between KSHV antibodies and parasite infections including *P*. *falciparum* and helminths (hookworm and *Mansonella perstans*) in rural [4] and peri-urban [20–22] populations in Uganda. The Lake Victoria island communities in East Africa are characterised by poor sanitation and a high prevalence of infectious diseases, including schistosomiasis [23–26]. No study to date has documented the burden of KSHV or KS in these unique communities. This study aimed to determine the seropositivity of KSHV in the Lake Victoria island communities of Koome sub-county, Uganda and the association between KSHV seropositivity and parasite co-infections.

## Methods

### Study population, participant selection and ethical approval

This cross-sectional study used samples from an open cluster-randomised trial of intensive versus standard anthelminthic treatment, the Lake Victoria Island Intervention Study on Worms and Allergy-related diseases (LaVIISWA) (ISRCTN47196031) [27]. The study was carried out in the Lake Victoria island communities of Koome sub-county, Mukono district, Uganda between September 2012 and August 2016. Twenty-six island villages were included in the trial. These 26 villages were randomised to receive either standard or intensive anthelminthic treatment, with 13 villages in each trial arm. Residents of villages in the standard arm were offered a single dose of albendazole (400mg) twice yearly and a single dose of praziquantel (40mg/Kg) annually. Residents of villages in the intensive arm received a triple dose of albendazole (400mgX3) and a single dose of praziquantel (40mg/Kg), given four times yearly. Details of the trial are published elsewhere [27, 28]. Two major separate surveys were carried out, one at baseline before the trial intervention (baseline survey) and the second after three years of the trial intervention (final survey). At baseline, a household survey was conducted where 32 to 45 households per village were selected at random to participate. After three years of the intervention, the final household survey was conducted, which involved selecting a random sample of 70 households per village. All residents aged one year and above of selected households were invited to participate. Separate random households were selected for each survey, without individual participant follow-up. Data on socio-demographic characteristics were collected, clinical examinations were performed, and blood and stool samples were taken. A total of 1310 (baseline survey) and 1571 (final survey) plasma samples were selected randomly for KSHV antibody testing. Ethical approvals were obtained from the Uganda Virus Research Institute Research and Ethics Committee (reference number: GC/127/17/04/317), the Uganda National Council for Science and Technology (reference number: HS1183) and the London School of Hygiene & Tropical Medicine Research and Ethics committee (reference number: 9917–9). Written informed consent was obtained from all adults aged 18 years and above. Children below 18 years were consented into the study by their parents or guardians; we also sought, in addition to parental consent, written assent from children aged between 8–17 years.

### Serology

IgG antibodies to K8.1 and ORF73 recombinat proteins were measured using ELISA to determine KSHV seropositivity, as previously described [29]. Each plate contained three negative and positive controls. The negative controls were used to determine the cut-off value. Seropositivity was defined as reactivity to either K8.1 or ORF73 antigens or both. This assay has been shown to be specific and sentitive to KSHV infection [29]. IgE, IgG (all subclasses) and IgG4 antibody concentrations to *Schistosoma mansoni* Egg Antigen (SEA) and *Schistosoma mansoni* adult Worm Antigen (SWA) were measured using ELISA. Antigen concentrations of 8 μg/mL (SWA) and 2.4 μg/mL (SEA) plus sample dilutions of 1/20 (IgE), 1/200 (IgG4) and 1/3000 (IgG) were used, as previously reported [14, 30, 31].

### Diagnostic testing and socio-demographic data collection

Duplicate slides were made from each stool sample and analysed independently by two technicians for helminth infection and intensity using the Kato-Katz (KK) method [32]. The following thresholds were used for classification of *S*. *mansoni* infection intensities: 1–99 eggs per gram of stool as light intensity, 100–399 eggs per gram of stool as moderate intensity and greater than 400 eggs per gram of stool as heavy intensity. An aliquot of the stool was stored in 70% ethanol and later used to detect helminths by real-time PCR. The multiplex real-time PCR assay was used to detect *Necator americanus*, *Strongyloides stercoralis* and *Schistosoma mansoni* as previously described [33, 34]. *S*. *mansoni* antigens were also tested in urine using the Circulating Cathodic Antigen (CCA) kits (Rapid Medical Diagnostics, Pretoria, South Africa) [27]. *Mansonella perstans* infection was detected using the modified Knott’s method [35] and thick blood films were made for malaria parasitaemia detection using microscopy. Questionnaires were administered for demographic data collection while HIV infection status was determined using rapid HIV diagnostic kits following the Uganda national HIV testing algorithm.

### Statistical analysis

Statistical analysis was carried out using STATA version 13 (StataCorp, College Station, Texas USA). The survey study design of the main trial was non self-weighting (because the number of households selected from each village were not dependant on village size, therefore households from smaller villages were more likely to be included in the survey than households from larger villages). To allow for this non self-weighting design and to ensure that our analyses are representative of the study area, we therefore took into account clustering within villages and applied village-level weights to allow for the different village sizes for all observational analyses [36]. Logistic regression (allowing for the survey design) was used to determine associations between risk factors and KSHV seropositivity. Linear regression (allowing for the survey design) was used to determine associations between risk factors and KSHV antibody levels. Although KSHV antibody levels did not attain normal frequency distributions, they were log_10_ transformed prior to linear regression modelling. For assessing the effect of intensive versus standard treatment, the analysis was done at the cluster level. The proportion of KSHV seropositive participants was calculated for each village, and the mean of these taken for the two trial arms. The risk ratio (RR) was then calculated by dividing the mean KSHV prevalence in the intensive arm by that in the standard arm, and a Taylor approximation was used to calculate a 95% confidence interval for this RR. The p-value was generated from a t-test comparing the village-level prevalences between the two arms. A similar approach was used to assess the effect of intensive versus standard treatment on KSHV antibody levels. A p-value of less than 5% was considered statistically significant. Multivariable models included age (grouped), sex, HIV status, *S*. *mansoni*, hookworm and malaria parasite infection. Participants not tested for HIV were also included in the analyses.

## Results

### Participants characteristics

Baseline and final survey results were analysed separately. The median age at baseline was 25 with an interquartile range (IQR) of 3 to 33 years. In the final survey, the median age was 24 years with an interquartile range (IQR) of 9 to 33 years. The overall proportion of males was 56% at baseline and 48% in the final survey. Details of the socio-demographic characteristics of the study population are shown in Table 1. At baseline, the HIV prevalence was 13% overall and 17% in those aged 13 years and above. In the final survey, around a quarter of participants (26%), mainly children, were not tested for HIV. There were 201 HIV seropositive individuals among 1229 tested (17% prevalence), with 103 participants confirmed to be on antiretroviral treatment (ART). At baseline, the malaria prevalence was 7% overall and 14% in children below 12 years. In the final survey, malaria infection prevalence was lower, at 4% overall, and 8% in children aged 1 to 12 years. Among the helminth infections tested, *Schistosoma mansoni* was the most prevalent in both surveys, as expected, due to the close proximity of the study sites to the waters of Lake Victoria. The prevalence was 86% by CCA, 50% by PCR and 34% by microscopy in the final survey (Table 1). At baseline, the prevalence was 72% by CCA in a subset of 569 participants and 52% by microscopy in 1137 participants. Hookworm prevalence was 7% by microscopy and 26% by PCR at baseline while in the final survey, it was 2% by microscopy and 8% by PCR. The prevalence of other helminths at baseline was 14% for *Strongyloides stercoralis* (using PCR), 11% for *Trichuris trichiura* (using KK), 0.1% for *Ascaris lumbricoides* (using KK) and 3% for *Mansonella perstans* (Table 1). While in the final survey, the prevalence of other helminths was 6% for *Strongyloides stercoralis* (using PCR), 9% for *Trichuris trichiura* (using KK), 0.04% for *Ascaris lumbricoides* (using KK) and 0.9% for *Mansonella perstans* (Table 1). At baseline, 20% of the *S. monsoni* infected individuals had light intensity infections, 15% moderate and 16% heavy infections. On the other hand, the majority of the infected individuals in the final survey had light to moderate helminth infections; 8% had a heavy *S*. *mansoni* infection based on microscopy (Table 1).

**Table 1:** Characteristics of the study population.

Factor	Baseline survey	Final survey
	n = 1310	n = 1571
Age, median and inter quartile range	25 (3–33)	24 (9–33)
Age group, years		
1–12	29% (362/1308)	31% (492/1571)
13–30	40% (546/1308)	39% (596/1571)
31–44	24% (319/1308)	22% (353/1571)
above 44	7% (130/1308)	9% (130/1571)
Sex (males)	56% (744/1310)	48% (801/1571)
HIV prevalence		
Overall	13% (145/1150)	17% (201/1229)
Participants aged 1–12 years	0.04% (1/288)	2% (6/270)
Participants aged 13 and above	17% (144/862)	21% (195/959)
ART status		
(+) treated		57% (103/201)
(+) untreated		6% (13/201)
(+) no treatment status		37% (85/201)
Malaria infection		
Overall	7% (92/1307)	4% (60/1554)
Children (1–12 years)	14% (51/361)	8% (34/491)
*Schistosoma mansoni* prevalence		
Microscopy (KK)	52% (606/1137)	34% (440/1355)
*PCR*		50% (673/1353)
CCA	72% (414/569)	86% (1225/1430)
*Schistosoma mansoni* intensity KK		
Uninfected	48% (531/1137)	66% (915/1355)
Light infection	20% (238/1137)	16% (216/1355)
Moderate	15% (183/1137)	10% (119/1355)
Heavy	16% (185/1137)	8% (105/1355)
*Necator americanus* prevalence PCR	26% (295/1136)	8% (120/1353)
Hookworm prevalence KK	7% (82/1137)	2% (30/1355)
*Strongyloides stercoralis* prevalence PCR	14% (176/1136)	6% (98/1353)
*Trichuris trichiura* prevalence KK	11% (148/1137)	9% (135/1355)
*Ascaris lumbricoides* prevalence KK	0.1% (15/1137)	0.04% (8/1355)
*Mansonella perstans* prevalence	3% (37/1296)	0.9% (14/1567)

Proportions were weighted to allow for the survey design and thus not calculated directly from the numerators and denominators presented in the table. Helminths infection status and were determined from a single stool sample using Kato-Katz (KK) method or PCR (polymerase chain reaction) method or both. Rapid tests were used for HIV screening and microscopy was used for malaria diagnosis.

### KSHV seropositivity

**Fig 1:**
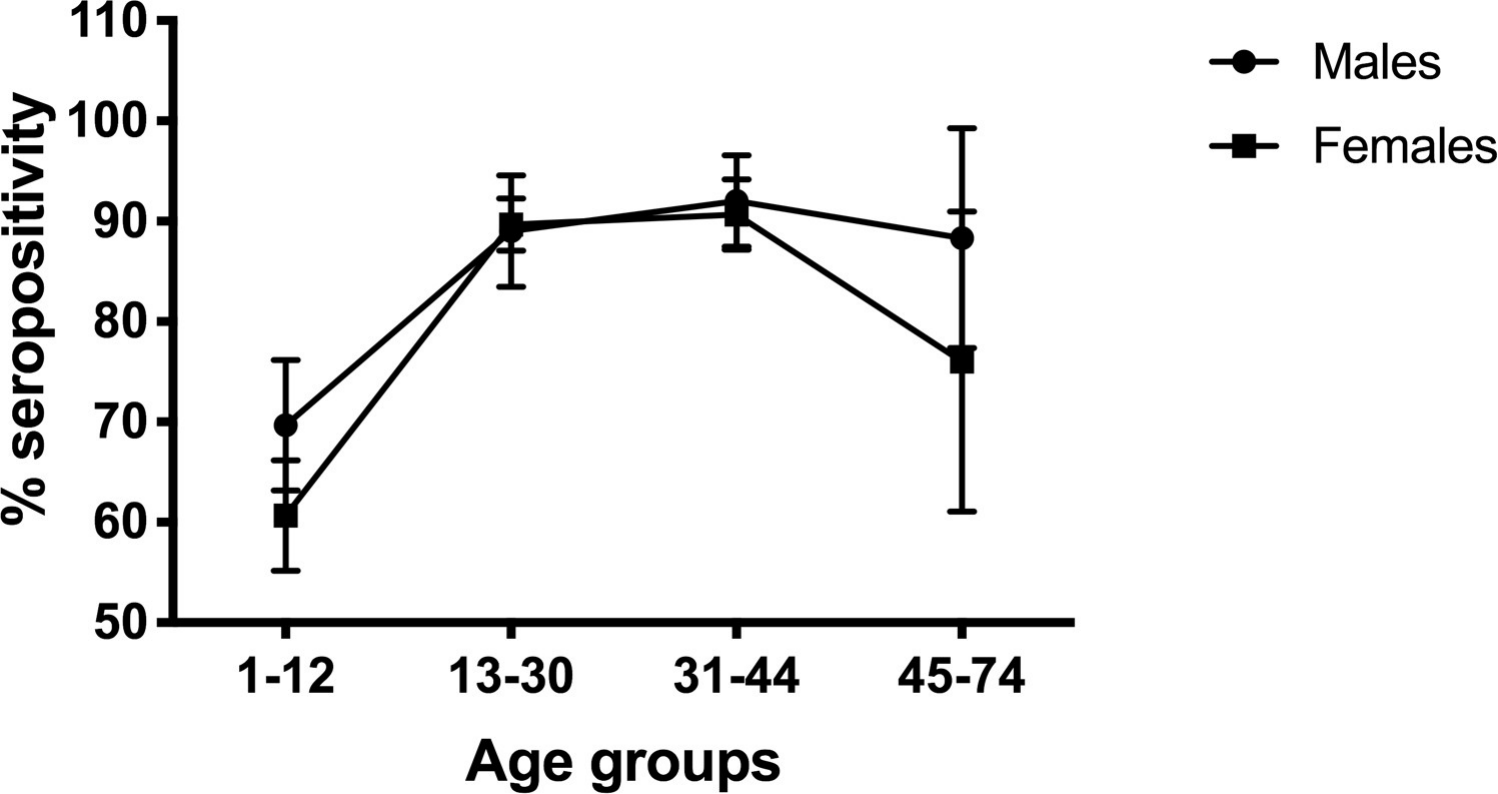
KSHV seropositivity and 95% confidence intervals (CI) across ages 1 to 74 years in the baseline survey. KSHV Seropositivity defined as reactivity to either ORF73 or K8.1 proteins. KSHV antibodies were detected using ELISA. Seropositivity and 95% CI were obtained in STATA, allowing for the survey design.

**Fig 2:**
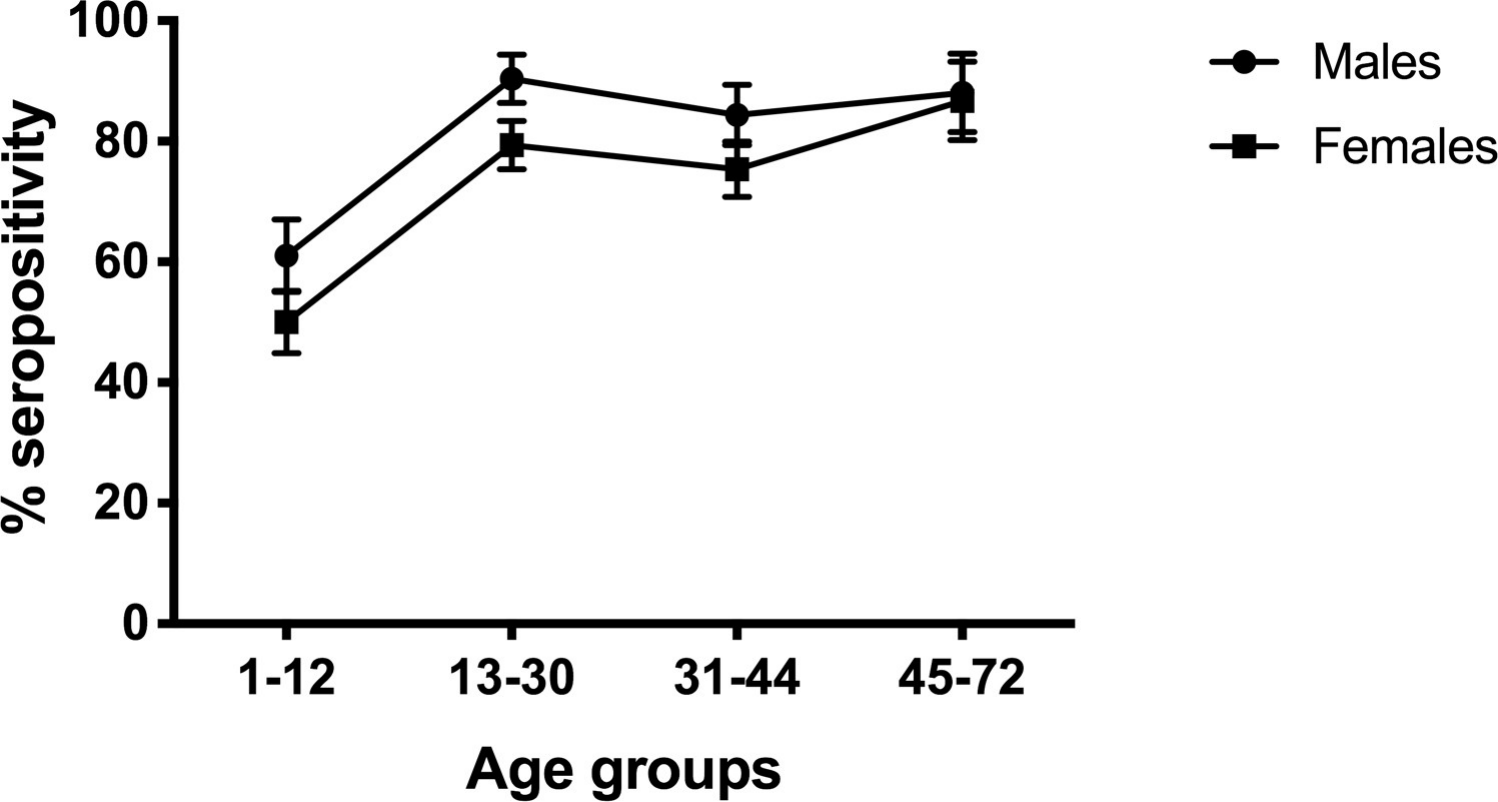
KSHV seropositivity and 95% confidence intervals (CI) across ages 1 to 72 years in the final survey. KSHV Seropositivity defined as reactivity to either ORF73 or K8.1 proteins. KSHV antibodies were detected using ELISA. Seropositivity and 95% CI were obtained in STATA, allowing for the survey design.

Overall KSHV seropositivity was 84% at baseline and 75% in the final survey. At baseline, the prevalence increased from 66% at 1–12 years to 93% in 31–44 years and 90% in 45-74-year-olds (Fig 1). In the final survey, the prevalence increased steeply with age (overall p<0.0001), rising from 56% in the 1-12-year age group to 84% in the 13-30-year age group, and plateaued thereafter (Fig 2).

### Associations between KSHV seropositivity and risk factors

We investigated associations of KSHV seropositivity with parasite infections and other factors at baseline and in the final survey. Overall, KSHV prevalence was higher in males compared to females (adjusted Odds Ratio (aOR) = 1.72 (1.29, 2.30), p = 0.001) in the final survey (Table 2). HIV seropositive individuals on ART were less likely to be KSHV seropositive compared to HIV seronegative individuals in the final survey (Table 2).

**Table 2:** Association between KSHV seropositivity and risk factors in the final survey.

Factor	KSHV seropositivity	Crude OR^a^ (95% CI^b^)	P value	Adjusted^c^ OR (95% CI)	P value
Age group (years)					
1–12	56% (289/492)	1		1	
13–30	84% (511/596)	4.35 (2.80,6.74)		4.84 (2.92, 8.02)	
31–44	81% (294/353)	3.44 (2.28, 5.19)		4.13 (2.40, 7.10)	
45–72	89% (113/130)	6.65 (3.00,14.77)	<0.0001	7.74 (3.47, 17.27)	<0.0001
Sex					
Female	71% (561/770)	1		1	
Male	80% (646/801)	1.68 (1.26, 2.25)	0.001	1.72 (1.29, 2.30)	0.001
HIV status					
Negative	79% (813/1028)	1		1	
(+) treated	70% (71/103)	0.64 (0.47, 0.87)		0.46 (0.30, 0.71)	
(+) untreated	76% (10/13)	0.88 (0.22, 3.50)		0.55 (0.14, 2.16)	
(+) no treatment status	85% (73/85)	1.60 (0.82, 3.11)		1.22 (0.57, 2.26)	
Not tested	67% (240/342)	0.55 (0.31, 1.00)	0.002	1.05 (0.56, 1.95)	0.002
*S*. *mansoni*					
Uninfected	73% (676/915)	1		1	
Infected	80% (359/440)	1.55 (1.13, 2.11)	0.008	1.43 (1.04, 1.95)	0.028
*N*. *americanus*					
Uninfected	74% (927/1233)	1		1	
Infected	86% (106/120)	2.15 (1.18, 3.94)	0.015	1.55 (0.86, 2.80)	0.136
*T*. *trichiura*					
Uninfected	75% (924/1220)	1		1	
Infected	79% (111/135)	1.25 (0.66, 2.39)	0.480	1.60 (0.83, 3.08)	0.150
*S*. *stercoralis*					
Uninfected	74% (949/1255)	1		1	
Infected	85% (84/98)	1.82 (0.72, 4.62)	0.198	1.03 (0.41, 2.61)	0.947
Malaria					
Negative	75% (1142/1494)	1		1	
Positive	84% (50/60)	1.76 (0.81, 3.82)	0.144	3.49 (1.08, 11.28)	0.038

Seropositivity defined as reactivity to either ORF73 or K8.1 proteins. KSHV antibodies were detected using ELISA. Rapid tests were used to determine HIV status. Statistical analysis was performed using logistic regression, allowing for the survey design. *Schistosoma mansoni* and *Trichuris trichiura* infections were determined from a single stool sample using the Kato-Katz method. *Necator americanus* and *Strongyloides stercoralis* infections determined using PCR (polymerase chain reaction) method.

^a^OR: odds ratios.

^b^CI: Confidence Intervals.

^c^ adjusted for age, sex, HIV status, *S*. *mansoni*, *N*. *americanus* and malaria parasitaemia. Proportions were weighted to allow for the survey design and thus not calculated directly from the numerators and denominators presented in the table.

Individuals infected with malaria parasites (aOR = 3.49 (1.08, 11.28), p = 0.038) were more likely to be KSHV seropositive in the final survey (Table 2). Although in the final survey, hookworm infection was positively associated with KSHV seropositivity in the unadjusted analysis (OR = 2.15 (1.18, 3.94), p = 0.015), this association was lost after adjusting for age group, sex, HIV serostatus, *S*. *mansoni* infection status and malaria infection status (Table 2). Helminth infections including *Trichuris trichiura* and *Strongyloides stercoralis* showed no association with KSHV seropositivity both at baseline and in the final survey (Table 3 & Table 2). Other helminth infections such as *Ascaris lumbricoides* and *Mansonella perstans* were not analysed using regression modelling due to the small numbers of infected participants.

**Table 3:** Association between KSHV seropositivity and risk factors at baseline.

Factor	KSHV seropositivity	Crude OR^a^ (95% CI^b^)	P value	Adjusted^c^ OR (95% CI)	P value
Age group (years)					
1–12	66% (236/362)	1		1	
13–30	90% (490/546)	4.65 (2.88,7.52)		5.14 (2.92, 9.05)	
31–44	93% (290/319)	6.50 (3.16, 13.36)		7.59 (3.31, 17.40)	
45–74	90% (74/81)	4.21 (1.41,12.57)	<0.0001	6.29 (2.54, 15.58)	<0.0001
Sex					
Female	81% (450/566)	1		1	
Male	86% (641/744)	1.41 (1.03, 1.94)	0.035	1.01 (0.63, 1.59)	0.980
HIV status					
Negative	84% (846/1005)	1		1	
Positive	89% (126/145)	0.65 (0.79, 3.45)	0.170	0.72 (0.36, 1.47)	0.357
*S*. *mansoni*					
Uninfected	77% (410/531)	1		1	
Infected	89% (533/606)	2.25 (1.45, 3.50)	0.001	1.86 (1.16, 2.99)	0.012
*N*. *americanus*					
Uninfected	82% (684/841)	1		1	
Infected	86% (258/295)	1.32 (0.92, 1.90)	0.125	1.21 (0.68, 2.15)	0.499
*T*. *trichiura*					
Uninfected	83% (816/989)	1			
Infected	86% (127/148)	1.28 (0.62, 2.67)	0.491		
*S*. *stercoralis*					
Uninfected	82% (786/960)	1		1	
Infected	89% (156/176)	1.77 (1.08, 2.89)	0.025	0.92 (0.59, 1.44)	0.708
Malaria					
Negative	84% (1016/1215)	1		1	
Positive	80% (72/92)	1.80 (0.51, 1.25)	0.310	1.27 (0.69, 2.33)	0.428

Seropositivity defined as reactivity to either ORF73 or K8.1 proteins. KSHV antibodies were detected using ELISA. Rapid tests were used to determine HIV status. Statistical analysis was performed using logistic regression, allowing for the survey design. *Schistosoma mansoni* and *Trichuris trichiura* infections were determined from a single stool sample using the Kato-Katz method. *Necator americanus* and *Strongyloides stercoralis* infections determined using PCR (polymerase chain reaction) method.

^a^OR: odds ratios.

^b^CI: Confidence Intervals.

^c^ adjusted for age, sex, HIV status, *S*. *mansoni*, *N*. *americanus* and malaria parasitaemia. Proportions were weighted to allow for the survey design and thus not calculated directly from the numerators and denominators presented in the table.

Individuals who were microscopy positive for *S*. *mansoni* at baseline (aOR = 1.86 (1.16, 2.99) p = 0.012 (Table 3) and in the final survey (aOR = 1.43 (1.04, 1.95), p = 0.028) (Table 2) were more likely to be KSHV seropositive. KSHV seropositivity increased with increasing *S*. *mansoni* infection intensity at baseline (p for trend = 0.013) (Table 4). In the final survey, the seropositivity of KSHV among individuals heavily infected with *S*. *mansoni* was 82% compared to 80% among lightly or moderately infected individuals, and 73% among uninfected individuals, although evidence for an increasing prevalence with increasing intensity was borderline after adjusting for possible confounders (P value for trend = 0.068) (Table 5). We did not observe significant associations between *S*. *mansoni* detected by CCA or PCR and KSHV seropositivity (S1 Text and S2 Text). There was no effect of intensive versus standard anthelminthic treatment on either KSHV seropositivity or antibody levels (Table 6).

**Table 4:** Associations between KSHV seropositivity and *S*. *mansoni* infection intensity at baseline.

Risk factor	KSHV seropositivity	Univariate		Age, sex, HIV, *N*. *americanus* and malaria adjusted	
		OR (95% CI)	P value	OR (95% CI)	P value
*S*. *mansoni* intensity					
Uninfected	77% (410/531)	1		1	
Light	87% (203/238)	1.91 (0.93, 3.92)		1.56 (0.79, 3.08)	
Moderate	91% (163/183)	3.00 (1.42, 6.37)		2.76 (1.17, 6.52)	
Heavy	88% (167/185)	2.20 (1.27, 3.81)	<0.0001 trend	1.66 (0.84, 3.26)	0.013 trend

KSHV Seropositivity defined reactivity to either ORF73 or K8.1 proteins. KSHV antibodies detected using ELISA. OR: odds ratios. *Schistosoma mansoni* was determined from a single stool sample using the Kato-Katz method. Statistical analysis was performed using logistic regression, allowing for the survey design. Proportions were weighted to allow for the survey design and thus not calculated directly from the numerators and denominators presented in the table.

**Table 5:** Associations between KSHV seropositivity and *S*. *mansoni* infection intensity in the final survey.

Risk factor	KSHV seropositivity	Univariate		Age, sex, HIV, *N*. *americanus* and malaria adjusted	
		OR (95% CI)	P value	OR (95% CI)	P value
*S*. *mansoni* intensity					
Uninfected	73% (676/915)	1		1	
Light	80% (173/216)	1.53 (1.14, 2.05)		1.38 (0.98, 1.93)	
Moderate	80% (99/119)	1.48 (0.94, 2.33)		1.29 (0.79, 2.11)	
Heavy	82% (87/105)	1.68 (0.71, 3.98)	0.049 trend	1.74 (0.69, 4.36)	0.068 trend

KSHV Seropositivity defined reactivity to either ORF73 or K8.1 proteins. KSHV antibodies detected using ELISA. OR: odds ratios. *Schistosoma mansoni* was determined from a single stool sample using the Kato-Katz method. Statistical analysis was performed using logistic regression, allowing for the survey design. Proportions were weighted to allow for the survey design and thus not calculated directly from the numerators and denominators presented in the table.

**Table 6:** Effect of helminths treatment on KSHV seropositivity and antibody levels.

		KSHV seropositivity				K8.1				ORF73			
	KSHV seropositivity	Crude RR^a^ (95% CI^b^)	P value	Adjusted RR (95% CI)	P value	Crude diff^c^ (95% CI)	P value	Adjusted diff (95% CI)	P value	Crude diff (95% CI)	P value	Adjusted diff (95% CI)	P value
Trial arm													
Standard	78% (550/710)	1		1		Ref		Ref		Ref		Ref	
Intensive	77% (657/861)	0.99 (0.92, 1.05)	0.690	1.00 (0.93, 1.07)	0.925	0.03 (-0.09, 0.15)	0.644	-0.06 (-0.18, 0.67)	0.352	0.06 (-0.06, 0.15)	0.312	-0.08 (-0.22, 0.06)	0.228

KSHV Seropositivity defined as reactivity to either ORF73 or K8.1 proteins. KSHV antibodies were detected using ELISA.

^**a**^RR: risk ratio.

^**b**^CI: confidence Interval.

^**c**^Diff: difference. Adjusted for sex, age group and HIV status. Proportions were weighted to allow for the survey design and thus not calculated directly from the numerators and denominators presented in the table.

### Association between KSHV antibody levels and microscopy status of *S*. *mansoni* infection

We further investigated the association between *S*. *mansoni* infection and infection intensity detected by microscopy and KSHV IgG antibody levels to K8. 1 and ORF73 separately. At baseline, microscopy positive individuals for *S*. *mansoni* had higher antibodies to K8.1 compared to microscopy negative individuals (adjusted linear regression coefficient = 0.03 (0.01, 0.05) p = 0.02). Furthermore, this association increased with increasing infection intensity (p value for trend = 0.005; S3 Text). Although antibody levels to ORF73 were not associated with *S*. *mansoni* infection at baseline, there was an association between ORF73 antibodies and *S*. *mansoni* infection intensity (p-value for trend = 0.026). Antibody levels to both K8.1 and ORF73 were neither associated with *S*. *mansoni* infection nor with *S*. *mansoni* infection intensity in the final survey (S3 Text).

### Associations between KSHV seropositivity and *Schistosoma mansoni* antibody concentrations

IgE (n = 364), IgG (n = 372) and IgG4 (n = 370) antibody concentrations against *S*. *mansoni* egg and adult worm antigens were measured from the final survey in a subset of individuals with sufficient plasma for the analysis. Participants whose samples were used for this analysis were on average older than participants whose samples were not used; other participant characteristics were comparable (S4 Text). After adjusting for age group, sex and HIV status, increased levels of IgE to SWA (aOR = 55.03 (3.14, 963.65), p = 0.008) and SEA (aOR = 8.20 (1.53, 44.05), p = 0.016) as well as IgG to SEA (aOR = 2.57 (1.17, 5.68), p = 0.02) were associated with an increased risk of being KSHV seropositive (Table 7).

**Table 7:** Associations between KSHV seropositivity and *Schistosoma mansoni* antibody concentrations.

Antibody type	Univariate		Age, sex and HIV adjusted	
	OR (95% CI)	P value	OR (95% CI)	P value
IgE to SEA (n = 364)	7.57 (1.54, 37.20)	0.015	8.20 (1.53, 44.05)	0.016
IgE to SWA (n = 364)	83.03 (4.69, 1470.38)	0.004	55.03 (3.14, 963.65)	0.008
IgG to SEA (n = 372)	3.03 (1.19, 7.77)	0.023	2.57 (1.17, 5.68)	0.021
IgG to SWA (n = 372)	6.99 (1.24, 39.49)	0.029	4.22 (0.98, 18.18)	0.053
IgG4 to SEA (n = 370)	1.30 (1.03, 1.62)	0.026	1.23 (0.96, 11.58)	0.097
IgG4 to SWA (n = 370)	1.37 (0.96, 1.97)	0.080	1.18(0.82, 1.71)	0.362

KSHV Seropositivity defined reactivity to either ORF73 or K8.1 proteins. KSHV and *Schistosoma mansoni* antibodies detected using ELISA, measured in ng/mL and converted to μg/mL. OR: odds ratios per unit increase in antibody level. Statistical analysis was performed using logistic regression, allowing for the survey design. SEA: *Schistosoma mansoni* Egg Antigen; SWA: *Schistosoma mansoni* Worm antigen. Ig: Immunoglobulin

## Discussion

KSHV prevalence can vary, even between geographically proximate areas [3]. We have previously reported a high KSHV seroprevalence of >95% in adults in the General Population Cohort in rural southwestern Uganda [5], and a prevalence of 61% amongst mothers in a peri-urban cohort [37]. This current study shows a high seropositivity of KSHV (>80% in 13+-year-olds) amongst Lake Victoria island communities with seropositive participants as young as one year. Additionally, we show that males were more likely to be KSHV seropositive compared to females. These findings are similar to those documented in other studies carried out in sub-Saharan Africa [4, 5, 38].

The HIV prevalence in the studied communities was high (17%). We observed a lower risk of being KSHV seropositive among individuals treated for HIV compared to HIV negative individuals. Since our study was cross-sectional and given the fact that there was missing HIV data, this finding should be treated with caution. However, ART has been shown to lead to tumour regression among AIDS-KS patients [39–41]. Additionally, others have shown a decline in KSHV viral load following HAART initiation [42–44], perhaps suggesting a direct effect of ART on KSHV replication.

The high untreated HIV prevalence, coupled with other factors including parasite infections, may contribute to the high prevalence of KSHV in this area. The burden of *S*. *mansoni* in these island communities is very high. We showed that being infected with *S*. *mansoni*, detected by microscopy, was associated with an increased risk of being KSHV seropositive. Others have reported no association between *S*. *mansoni* and KSHV infections, possibly due to the low prevalence of KSHV or low infection intensity of *S*. *mansoni* in the study areas [45, 46]. *In vitro* reactivation of the model gammaherpesvirus MHV68 by *S*. *mansoni* was demonstrated by Reese *et al*., mediated through IL4 production [18]. Our human data are consistent with this model, as we observed an association with *S*. *mansoni* and KSHV seropositivity. Furthermore, at baseline (before antihelminthic treatment), anti-K8.1 antibody levels were higher in *S*. *mansoni* infected individuals compared to uninfected individuals, and *S*. *mansoni* infection intensity was associated with higher antibody levels to K8.1 and ORF73. These associations were not observed in the final survey after antihelminthic treatment. Nonetheless, higher antibody levels to KSHV antigens, and particularly the lytic antigen K8.1, has been associated with viral reactivation and KS disease progression [47–50]. Our findings may imply KSHV reactivation in individuals with a heavy *S*. *mansoni* infection, although a major limitation in interpretation is that we are unable to assign causation. An additional limitation is that we did not measure KSHV viral load in blood due to the unavailability of appropriate samples, and this would reaffirm the role of *S*. *mansoni* infection in KSHV reactivation.

We also showed that increasing IgE and IgG, but not IgG4 antibodies to *S*. *mansoni* are associated with an increased risk of being KSHV seropositive in older individuals. The (Th) 2 immune response to *S*. *mansoni*, characterised by IgE, IL4 and IL5 production has been linked to protection from *S mansoni* reinfection, while IgG4 production has been linked to susceptibility to reinfection [51]. This protective immunity tends to increase with age and requires repeated exposure to develop. This would then suggest that *S*. *mansoni*, through (Th) 2 immune response upregulation, may reactivate KSHV latently infected cells.

Alternatively, the association between KSHV seropositivity and *S*. *mansoni* infection could be caused by increased inflammation due to new *S*. *mansoni* infections, leading to an increase in KSHV antibody levels in individuals already infected with KSHV. However, the association with anti-K8.1 (a lytic antigen) but not anti-ORF73 (a latent antigen) antibodies with *S*. *mansoni* infection at baseline may imply specific effects of *S*. *mansoni* on KSHV reactivation as opposed to non-specific inflammatory effects. Furthermore, higher anti-Ag85A (a *Mycobacteria tuberculosis* antigen unrelated to *S*. *mansoni*) IgG4, but not IgG, has been reported in *S*. *mansoni* infected individuals compared to *S*. *mansoni* uninfected individuals [14]. Taken together, this might suggest a specific effect of *S*. *mansoni* on immunity to other unrelated chronic infections.

The association between *S*. *mansoni* and KSHV seropositivity was only observed if *S*. *mansoni* was detected using microscopy (KK) but not PCR or CCA, in this study. Microscopic examination of faecal samples is the WHO recommended diagnostic test for *S*. *mansoni* [52, 53], and has a documented specificity of 100%, although varying sensitivity (depending on infection intensity and studied population) has been observed [53, 54]. Detection of *S*. *mansoni* by CCA may be influenced by cross-reactivity, leading to false positives, and may explain the larger numbers diagnosed by this method in our study [53, 54]. The PCR technique has been shown to be sensitive and specific for detecting *S*. *mansoni* [52–54]. The ability of the PCR technique to detect low-intensity *S*. *mansoni* infection, as well as the reduced infection intensity in the final survey due to the three years antihelminthic treatment, may have obscured the effect that *S*. *mansoni* has on KSHV antibody levels. Heavy *S*. *mansoni* infections might have a larger effect which could easily be observed with the current sample size. Furthermore, a light infection intensity could have effects on KSHV incident cases, as opposed to prevalent cases. Because of the observational study design, incident cases were not specifically detected, and our study likely picked up prevalent cases.

We did not see any effect of intensive versus standard anthelminthic treatment on KSHV seropositivity. This was not surprisingly as such an intervention would likely affect incident KSHV infections, rather than prevalent KSHV infections and antibody titres against KSHV would be unlikely to change over the short follow-up period. Migration rates in these Island communities were very high. Therefore very few people were tested both at baseline and in the final survey. Consequently, the majority of the participants analysed in the final survey might have been new immigrants. The original trial intended to obtain community/population-wide effects as opposed to individual participant effects.

We also found malaria parasitemia to be associated with KSHV seropositivity, consistent with our previous findings [4, 21], and these data reinforce the need to investigate the mechanism through which malaria infection impacts on KSHV and its subsequent role in KSHV transmission. Infection with *Plasmodium falciparum*, the main cause of malaria disease in Africa, induces inflammation which is normally regulated through induction of regulatory T cells and production of IL10 and TGF-β. Increased IL10 levels, a cytokine mainly produced by regulatory cells, has been reported in disseminated KS [55]. Plasmodia have also been shown to cause macrophage and dendritic cell dysfunction [56]. The immunosuppression caused by *P*. *falciparum* infection has also been shown to lead to the reactivation of some herpesviruses such as EBV, HSV-1 and VZV [57–61]. We therefore hypothesize that the immune dysregulation caused by malaria infection contributes to frequent reactivation of KSHV from latency. Since KSHV is transmitted by salivary exchange [3, 62, 63], studies examining parasite co-infections and KSHV viral load in saliva are warranted.

## Supporting information

S1 Text Association between KSHV seropositivity and *S*. *mansoni* by CCA method in the baseline survey. KSHV seropositivity defined reactivity to either ORF73 or K8.1 protein. KSHV antibodies detected using ELISA. ^a^OR: odds ratios. ^b^CI: confidence intervals. ^c^CCA (circulating cathodic antigen). Statistical analysis was performed using logistic regression, allowing for the survey design.
(DOCX)

S2 Text Association between KSHV seropositivity and *S*. *mansoni* by PCR and CCA methods in the final survey. KSHV seropositivity defined reactivity to either ORF73 or K8.1 protein. KSHV antibodies detected using ELISA. ^a^OR: odds ratios. ^b^CI: confidence intervals. ^c^CCA (circulating cathodic antigen). ^d^PCR: polymerase chain reaction. Statistical analysis was performed using logistic regression, allowing for the survey design.
(DOCX)

S3 Text Associations between KSHV antibodies and *S*. *mansoni* infection as well as infection intensity. KSHV antibodies were detected using ELISA. Statistical analysis was performed using linear regression, allowing for the survey design. *Schistosoma mansoni* was determined from a single stool sample using Kato-Katz method. ^a^Coef.: linear regression coeffient. ^b^CI: Confidence Intervals. ^c^ adjusted for age, sex, HIV status, *S*. *mansoni*, *N*. *americanus* and malaria parasiteamia.
(DOCX)

S4 Text Infection status and study characteristics of participants tested for *Schistosoma mansoni* antibody responses compared to those not tested. P value obtained from a Chi^2^ test, allowing for the survey design.
(DOCX)
