# Supplementary material for: Kaposi’s sarcoma-associated herpesvirus seropositivity is associated with parasite infections in Ugandan fishing communities on Lake Victoria islands
Source: PLoS Negl Trop Dis. 2019 Oct 16;13(10):e0007776. doi: 10.1371/journal.pntd.0007776 (PMC6816576; doi:10.1371/journal.pntd.0007776)
Supplement: S2 Text — KSHV seropositivity defined reactivity to either ORF73 or K8.1 protein. KSHV antibodies detected using ELISA. aOR: odds ratios. bCI: confidence intervals. cCCA (circulating cathodic antigen). dPCR: polymerase chain reaction. Statistical analysis was performed using logistic regression, allowing for the survey design. (DOCX) [file pntd.0007776.s002.docx]

S2 Text: Association between KSHV seroprevalence and *S. mansoni* by PCR and CCA methods in the final survey

| Risk factor | KSHV seroprevalence | Crude | | Age, sex, HIV,  *N. americanus* and malaria adjusted | | |
| --- | --- | --- | --- | --- | --- | --- |
|  |  | OR (95% CI) | P value | OR (95% CI) | P value |  |
| *S. mansoni* (CCA)  Uninfected  Infected | 80% (160/205)  76% (955/1225) | 1  0.82 (0.50, 1.33) | 0.402 | 1  0.87 (0.46, 1.65) | 0.654 |  |
| *S.mansoni* (PCR)  Uninfected  Infected | 73% (501/680)  77% (532/673) | 1  1.25 (0.97, 1.63) | 0.083 | 1  1.16 (0.84, 1.62) | 0.351 |  |

KSHV seropositivity defined reactivity to either ORF73 or K8.1 protein. KSHV antibodies detected using ELISA. ^a^OR: odds ratios. ^b^CI: confidence intervals. ^c^CCA (circulating cathodic antigen). ^d^PCR: polymerase chain reaction. Statistical analysis was performed using logistic regression, allowing for the survey design. Proportions were weighted to allow for the survey design and thus not calculated directly from the numerators and denominators presented in the table.
