# Supplementary material for: Kaposi’s sarcoma-associated herpesvirus seropositivity is associated with parasite infections in Ugandan fishing communities on Lake Victoria islands
Source: PLoS Negl Trop Dis. 2019 Oct 16;13(10):e0007776. doi: 10.1371/journal.pntd.0007776 (PMC6816576; doi:10.1371/journal.pntd.0007776)
Supplement: S3 Text — KSHV antibodies were detected using ELISA. Statistical analysis was performed using linear regression, allowing for the survey design. Schistosoma mansoni was determined from a single stool sample using Kato-Katz method. aCoef.: linear regression coeffient. bCI: Confidence Intervals. c adjusted for age, sex, HIV status, S. mansoni, N. americanus and malaria parasiteamia. (DOCX) [file pntd.0007776.s003.docx]

S3 Text: Associations between KSHV antibodies and *S. mansoni* infection as well as infection intensity

| Baseline survey | | | | | | | | | | |
| --- | --- | --- | --- | --- | --- | --- | --- | --- | --- | --- |
|  | K8.1 | | | | | ORF73 | | | | |
|  | Univariate analysis | | Multivariate analysis^c^ | | | Univariate analysis | | Multivariate analysis^c^ | | |
|  | Coef^a^ (95% CI^b^) | P value | Coef (95% CI) | P value | | Coef (95% CI) | P value | Coef (95% CI) | | P value |
| *S. mansoni*  Uninfected  Infected | 0.05 (0.03, 0.08) | **<0.0001** | 0.03 (0.01, 0.06) | **0.02** | | 0.05 (0.02, 0.08) | **0.001** | 0.02 (-0.01, 0.05) | | 0.129 |
| S. mansoni intensity  Uninfected  Light infection  Moderate infection  Heavy infection | 0.03 (-0.01, 0.07)  0.09 (0.05, 0.12)  0.05 (0.02, 0.08) | **<0.0001**  trend | - 1. (-0.03, 0.05)   0.07 (0.04, 0.11)  0.03 (-0.002, 0.06) | **0.005**  trend | | - 1. (-0.07, 0.06)   0.08 (0.04, 0.11)  0.06 (0.02, 0.09) | **<0.0001**  trend | -0.001 (-0.03, 0.03)  0.06 (0.02, 0.97)  0.02 (-0.01, 0.05) | | **0.026**  trend |
| Final survey | | | | | | | | | | |
| *S. mansoni*  Uninfected  Infected | 0.01 (-0.1, 0.12) | 0.829 | -0.02 (-0.09, 0.05) | 0.486 | 0.07 (-0.03, 0.17) | | 0.134 | | 0.05 (-0.03, 0.13) | 0.149 |
| *S. mansoni* intensity  Uninfected  Light infection  Moderate infection  Heavy infection | - 1. (-0.12, 0.13)   0.05 (-0.07, 0.16)  -0.03 (-0.16, 0.10) | 0.918 | -0.02 (-0.12, 0.08)  0.003 (-0.07, 0.08)  -0.04 (-0.18, 0.09) | 0.508 | 0.08 (-0.02, 0.17)  0.11 (0.001, 0.22)  -0.003 (-0.10, 0.09) | | 0.312 | | - 1. (-0.04, 0.14)   0.09 (0.01, 0.16)  -0.002 (-0.09, 0.09) | 0.287 |

KSHV antibodies were detected using ELISA. Statistical analysis was performed using linear regression, allowing for the survey design. *Schistosoma mansoni* was determined from a single stool sample using Kato-Katz method. ^a^Coef.: linear regression coeffient. ^b^CI: Confidence Intervals. ^c^ adjusted for age, sex, HIV status, *S. mansoni*, *N. americanus* and malaria parasiteamia.
