# Supplementary material for: Kaposi’s sarcoma-associated herpesvirus seropositivity is associated with parasite infections in Ugandan fishing communities on Lake Victoria islands
Source: PLoS Negl Trop Dis. 2019 Oct 16;13(10):e0007776. doi: 10.1371/journal.pntd.0007776 (PMC6816576; doi:10.1371/journal.pntd.0007776)
Supplement: S4 Text — P value obtained from a Chi2 test, allowing for the survey design. (DOCX) [file pntd.0007776.s004.docx]

S4 Text: Infection status and study characteristics of participants tested for *Schistosoma mansoni* antibody responses compared to those not tested

|  | Tested (n=372) | Not tested (n=1199) | P value |
| --- | --- | --- | --- |
| Age groups  1-12  13-30  31-44  45-72 | 14%  52%  23%  11% | 36%  35%  22%  8% | <0.0001 |
| Sex  Male | 44% | 49% | 0.053 |
| HIV prevalence | 17% | 17% | 0.914 |
| Malaria parasitaemia | 4% | 4% | 0.515 |
| Schistosoma mansoni KK | 16% | 17% | 0.858 |

P value obtained from a Chi^2^ test, allowing for the survey design.
